# Care programs and their components for patients with idiopathic pulmonary fibrosis: a systematic review

**DOI:** 10.1186/s12931-021-01815-8

**Published:** 2021-08-16

**Authors:** Anouk Delameillieure, Sarah Vandekerkhof, Bastiaan Van Grootven, Wim A. Wuyts, Fabienne Dobbels

**Affiliations:** 1 Department of Chronic Diseases and Metabolism, Laboratory of Respiratory Diseases and Thoracic Surgery, KU Leuven, Leuven, Belgium; 2 Department of Public Health and Primary Care, Academic Centre for Nursing and Midwifery, KU Leuven, Kapucijnenvoer 35 blok D-box 7001, 3000 Leuven, Belgium; 3 Research Foundation-Flandres, Brussels, Belgium; 4 Department of Respiratory Diseases, Unit for Interstitial Lung Diseases, University Hospitals Leuven, Leuven, Belgium

**Keywords:** Idiopathic pulmonary fibrosis, Chronic Care Model, Systematic review, Processes of care

## Abstract

**Background:**

The multidimensional and complex care needs of patients with idiopathic pulmonary fibrosis (IPF) call for appropriate care models. This systematic review aimed to identify care models or components thereof that have been developed for patients with IPF in the outpatient clinical care, to describe their characteristics from the perspective of chronic integrated care and to describe their outcomes.

**Methods:**

A systematic review was conducted using state-of-the-art methodology with searches in PubMed/Medline, Embase, CINAHL and Web Of Science. Researchers independently selected studies and collected data, which were described according to the Chronic Care Model (CCM).

**Results:**

Eighteen articles were included describing 13 new care models or components. The most commonly described CCM elements were ‘delivery system design’ (77%) and ‘self-management support’ (69%), with emphasis on team-based and multidisciplinary care provision and education. The most frequently described outcome was health-related quality of life.

**Conclusions:**

Given the high need for integrated care and the scarcity and heterogeneity of data, developing, evaluating and implementing new models of care for patients with IPF and the comprehensive reporting of these endeavours should be a priority for research and clinical care.

**Supplementary Information:**

The online version contains supplementary material available at 10.1186/s12931-021-01815-8.

## Background

Progressive fibrosing interstitial lung diseases (PF-ILDs) embody a group of rare diseases affecting the parenchyma of the lungs due to the production of self-sustaining fibrosis, of which idiopathic pulmonary fibrosis (IPF) is the most common one [1, 2]. Individuals experience worsening respiratory symptoms, a decrease in quality of life and face a poor median survival of 2–5 years [3, 4]. Over the past years, the IPF landscape changed significantly as new evidence on epidemiology, the chronicity of the disease, the role of antifibrotic drugs in slowing down disease progression, the high costs of care and the high prevalence of multimorbidities (e.g., pulmonary hypertension or gastro-oesophageal reflux) accumulates [2, 4–10]. Also, increasing evidence on patients’ needs has broadened our knowledge about the impact of IPF on patients’ lives, whereby not only the physical aspects of life are affected, but also the psychosocial dimensions [11–15].

Because of the complexity of the disease, and in line with policy recommendations, including the European IPF patient charter, patients require long-term care from specialized healthcare professionals to address their multidimensional medical, pharmaceutical and psychosocial challenges [16]. One way to do this is by adopting an integrated model of care across the care continuum from diagnosis until death that addresses all patients’ needs, and emphasizes patient-centredness, as has been suggested by the World Health Organization (WHO) also [17–19].

Although integrated chronic care models might be valuable for IPF, research on effective chronic management models and their components, however, is scarce. Having an overview of existing chronic care models for patients with IPF will provide a useful guide to assist care programs in moving towards patient-centred and integrated care.

This systematic review aims to identify care models and components aiming to change care in the outpatient IPF care, and to describe their characteristics from the perspective of chronic integrated care.

## Methods

This systematic review followed the handbook of the ‘Centre for Reviews and Dissemination’ (CRD) and was registered in the PROSPERO database (ID#CRD42020148929) [20]. Additional file 1 contains the PRISMA checklist [21]. This systematic review was conducted between June 2019 (i.e., drafting of the protocol) and October 2020 (i.e., data extraction and analysis completed).

### Search strategy

We performed searches in four electronic bibliographic databases from inception to April 16th, 2020: Medline (PubMed interface), CINAHL, EMBASE and Web of Science. The search string was developed together with an information specialist, covering two groups of search terms related to the health condition (i.e., IPF) and care components. Although this review focuses on IPF, we also included terms referring to diseases that may be classified under the recently published PF-ILD umbrella, depending on whether a progressive fibrosing lung phenotype emerges [1, 4, 22]. The term PF-ILD is not yet widely used in routine practice. Hence, we kept the search strategy broad to identify any articles that investigated the care management of patients affected by a progressive fibrosis [23].

Search terms related to care components were based on key literature papers and Thesauri of bibliographic databases. We developed the string in Medline first (see Additional file 2 ‘Search string’) and adapted it to fit Embase, Cinahl and Web of Science. We also conducted a ‘related article search’ in Medline, browsed the reference lists of included studies for additional papers, and performed a ‘Google Scholar’ search on April 16th, 2020, screening the first 100 hits for the entry term ‘care models in idiopathic pulmonary fibrosis’.

### Inclusion- and exclusion criteria

Included studies had to meet all of the following criteria: (1) full papers written in Dutch, French or English; (2) containing original research data (all designs); (3) focusing on IPF; (4) targeting outpatient care for adults; and (5) describing or evaluating a change in care. For studies targeting multiple diseases, we only included studies in which ≥ 50% of the participants had PF-ILD or in which separate data for IPF were reported.

We excluded; (1) abstracts; (2) publications not containing original research findings (e.g., reviews, book chapters, letters to editors); or (3) studies focusing on the diagnostic phase of PF-ILD exclusively.

### Procedure

Records were retrieved in EndNote X9 and duplicates were removed using Bramer and colleagues’ recommendations [24]. The remaining records were uploaded in the review management tool “Rayyan QCRI” (http://rayyan.qcri.org) [25]. Pairs of researchers (FD, SV and AD) identified eligible papers in two consecutive phases. The title and abstract screening phase was followed by an assessment of the full texts of all records that was deemed ‘potentially eligible’ by at least one reviewer using a piloted eligibility checklist. The authors discussed conflicting findings until consensus was reached. For eight papers, we contacted the corresponding authors. Four authors provided additional information allowing us to determine eligibility. We excluded articles of which the authors had not responded (n = 4).

### Data extraction and synthesis

Given the clinical and methodological heterogeneity of included articles, we used narrative synthesis to describe our study findings, guided by the Cochrane guideline on data synthesis and analysis [26]. Also, due to this heterogeneity, it was not possible to calculate effect sizes.

Using data extraction sheets, one reviewer (AD) extracted the data from the publications and the second reviewer (FD) independently reviewed these data for accuracy. To facilitate data extraction and synthesis, studies were categorized in two groups, based on whether or not the described and/or tested care model or component was implemented in healthcare practice. Extracted data covered study characteristics as well as characteristics of the care model or component under investigation.

The unique care models or components were described using the Chronic Care Model (CCM). This framework for integrated care has been put forward to guide healthcare delivery improvements towards chronic and proactive care and showed positive outcomes in a number of chronic diseases [27–29]. Table 1 provides an overview of the definitions of the elements of the Chronic Care Model, i.e., delivery system design, self-management support, health organization, community resources, decision support and clinical information system.

**Table 1:** Overview of the definitions of the elements of the Chronic Care Model (CCM) [27]

Health organization	*“Create a culture, organization and mechanisms that promote safe, high quality care”*
Decision support	*“Promote clinical care that is consistent with scientific evidence and patient preferences”*
Delivery system design	*“Assure the delivery of effective, efficient clinical care and self-management support”*
Clinical information system	*“Organize patient and population data to facilitate efficient and effective care”*
Self-management support	*“Empower and prepare patients to manage their health and health care”*
Community	*“Mobilize community resources to meet needs of patients”*

Copyright 1996–2020 The MacColl Centre. The Improving Chronic Illness Care program is supported by The Robert Wood Johnson Foundation, with direction and technical assistance provided by Group Health's MacColl Centre for Health Care Innovation”, available from http://www.improvingchroniccare.org/

### Quality assessment

Two researchers (AD and FD) independently assessed the quality of the eligible articles using the Mixed Methods Appraisal Tool (MMAT) [30]. The MMAT allows a critical appraisal of various types of qualitative, quantitative or mixed-methods research designs. Each design is evaluated using five criteria that could be either fulfilled (‘yes’), unfulfilled (‘no’), or was not reported/not applicable (‘can’t tell’). No total score was calculated.

## Results

### Search strategy

**Fig. 1:**
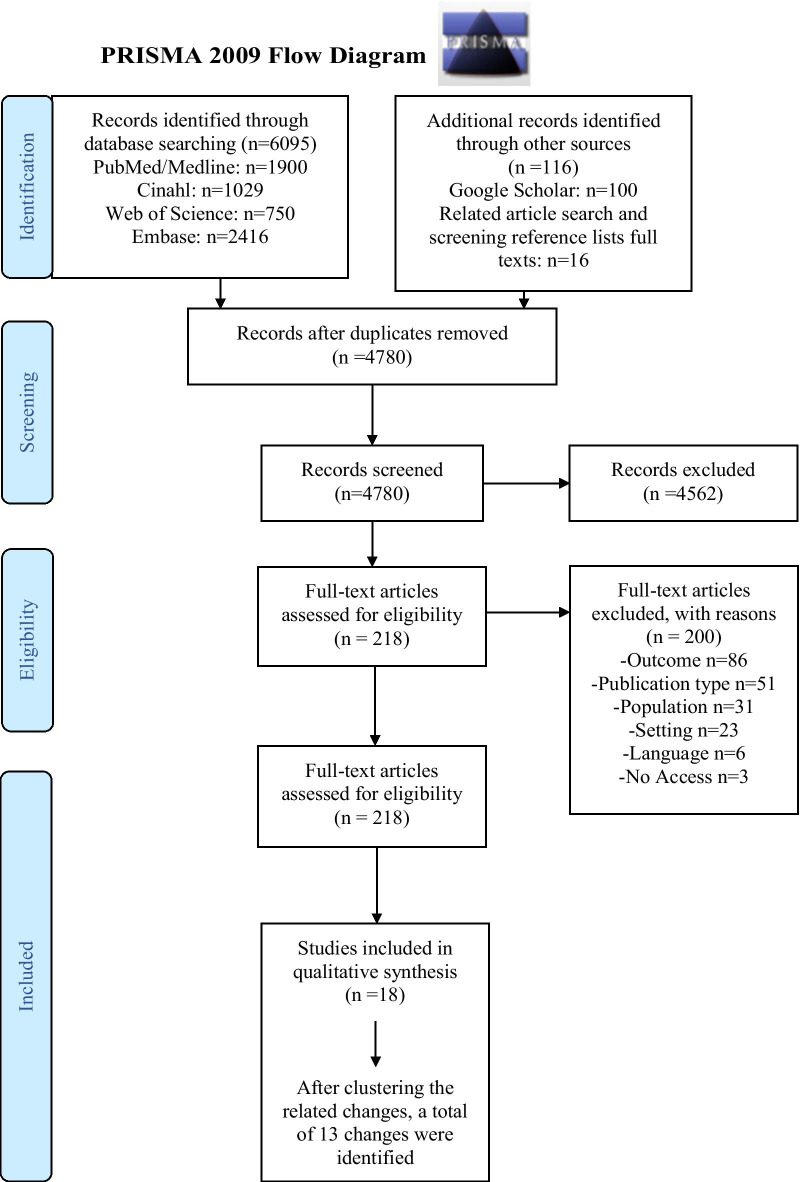
Prisma Flow Chart

Out of 4780 references, 18 articles were included (Fig. 1 Prisma Flow Chart). All reported on single centre studies. Eleven studies were based in Europe [i.e., the Netherlands (N = 3), UK (N = 5), Italy (N = 2) and Greece (N = 1)], two in the United States and five in Canada. Additional file 3 provides a detailed overview of the “study characteristics”. Seven studies were pilot or feasibility studies, six assessed the impact or effectiveness of the change, three articles solely described the changes in care and two used a quality improvement process to attain their change (Additional file 4 “Overview of the research phases”).

### Quality appraisal

Study designs were quantitative descriptive (N = 6), quantitative nonrandomized (N = 6), qualitative (N = 1) and three mixed methods studies. Also, we identified two case reports that were not appraised for quality.

One study did not provide a clear research question and only one article met all criteria reflecting good methodological quality (Additional file 5 “Quality assessment according to the MMAT”). All articles with a quantitative nonrandomized design missed information on the intended administration of the intervention and the inclusion of confounders in the analysis. All mixed-methods studies used an adequate rationale to combine a quantitative and qualitative part.

### Characteristics of the care model or component

Articles referring to the same change were clustered, resulting in 13 unique changes in a care model or component thereof. We refer the reader to Table 2 “Characteristics of the changes” and to the Additional file 6 “Overview of the characteristics of the identified care models and components” to find the characteristics of the care models or components. Seven were described or evaluated, but not yet routinely implemented and six were implemented in routine care and described and/or evaluated.

**Table 2:** Characteristics of the changes

Name of the program	What is the focus of change?	Which format is used?	What are the elements of the program?	Which main types of outcomes were assessed?
Overview of the care program or component thereof without implementation in routine clinical care yet (n = 7)				
Hospital2Home [31]	Palliative care	Nurse-led case conference	Multidisciplinary team-based care Lead of the conference: trained palliative care specialist nurse Involvement patient/caregiver in decision-making Situated in community setting Individual care plan and follow-up of action points	Primary outcomes: Palliative Care Outcome Scale Other outcomes: Patient-reported outcomes Feasibility Patient experiences
Aerodigestive multidisciplinary team [39]	Assessment co-morbidity	Multidisciplinary team meeting	Multidisciplinary team-based care Medical care	Clinical outcomes Feasibility
PRISIM [46]	Support- Coping	Group-based sessions	Psychoeducation 6-weeks program with two-hour group sessions	Patient-reported outcomes Patient experiences
Nurse-led support group [40]	Support- Advocacy group	Support group	Patient advocacy/support group (two-hour meetings once a month) Lead of the group: nurse	Patient-reported outcome
IPF online [37, 45]	Use of eHealth in care	eHealth platform	eHealth personal platform including information, PROMs, medication use, individual results of lung function tests and medication coach eConsult possibility Home-based spirometry function	Feasibility and safety Patient-reported outcomes Patient experiences
MBSR [36]	Support- Coping	Mindfulness-based stress reduction program (group-based sessions)	Standardised mindfulness training (eight weekly group sessions and further training at home) Use of techniques such as the body scan, sitting mediation and light yoga Sessions provided by a MBSR instructor	Primary outcome: safety Patient-reported outcomes Feasibility
PPEPP [32]	Support- Coping	Group-based sessions	Psychoeducation (three group sessions) Lead of the sessions: psychologist Contributions to the sessions by pulmonologist, a nurse specialized in ILD, an oxygen supplier, a social worker and physiotherapists	Patient-reported outcomes Patient satisfaction
Overview of the care programs or components thereof implemented in routine care (n = 6)				
SCDAT Collaborative MDT meeting [33, 34]	Palliative care and advanced care planning	Tool for the assessment of needs Multidisciplinary team meeting	Tool used by clinicians in outpatient setting to assess patients’ needs Follow-up multidisciplinary team-based care (palliative care consultant, palliative care nurse, psychologist, ILD consultant, ILD nurse, pharmacist and MDT coordinator)	Process measures Stakeholders’ feedback
NPP [41]	Pharmacological management program	Follow-up visits	Nurse-led support of pharmacological needs	Clinical outcomes (description)
IPF care [43]	Pharmacological management program	Nurse-led telephone program	UK program: program led by nurses specialized in ILD, telephone contact Austria program: program led by nurses specialized in ILD, telephone contact and home visit	Clinical outcomes Patient satisfaction Feasibility
An educational initiative: performance improvement study [35]	Overall organisation of the care program	Follow-up team-based care	Performance improvement initiative: An educational initiative to improve team-based care in which metrics (quality indicators) are used to assess, measure and adapt the delivered care	Performance indicators (process measures) Stakeholders’ experiences
Use of care coordinator [42]	Overall organisation of the care program	Follow-up care with coordinator	Case coordination (similar to specialist IPF nurse): assessment and administration, patient education, discussing transplantation, drug reimbursements, discussing drugs, oxygen therapy, discussing tests and results	Patient-reported outcomes Patient satisfaction Process measures Economic analysis
MDC care model [38, 44]	Palliative care and advanced care planning	Multidisciplinary collaborative care model	Collaborative multidisciplinary team-based care Involvement patient/caregiver in decision-making Individual care plan and follow-up of action points Close link with community	Healthcare use Preferred place of death Caregivers’ experiences

### How were care models/components developed?

Only two of the 13 changes reported the use of a theoretical framework (i.e., the Medical Research Council guidance (MRC) on developing complex interventions) or a theoretical model (i.e., the stress-coping model) [31, 32]. Furthermore, two of the 13 changes used a stepwise process, such as the PDCA cycles or the DMAIC method, of which the first step was the analysis of local practice patterns and needs to inform the content of the change [33–35]. Three changes were developed based on existing interventions or tools from other research fields [31, 34, 36]. Stakeholder and expert involvement was cited in six of the 13 changes, including patients in two studies [32, 37]. For five changes, the development was not reported [38–42].

### What was the main focus of the changes?

Of the six changes already implemented, two focused on the overall organization of the care program. One change focused on the role and impact of a care coordinator in the management of IPF patients [42]. The second one referred to a performance improvement initiative aiming to optimize overall team-based care [35]. Two articles described the experiences with their pharmacological management program, and both emphasized the role of a nurse specialized in IPF/ILD [41, 43]. Lastly, we identified two changes with a specific focus on advanced care planning and palliative care needs. The first involved a new multidisciplinary collaborative care model for addressing the palliative care needs of patients and their caregivers with a specific emphasis on patient-centredness [38, 44]. The second change comprised of a tool to support care providers in addressing palliative care needs of patients in routine care and the practice was subsequently extended with a multidisciplinary meeting in which tailored referrals were implemented for patients identified with the tool [33, 34].

Of the seven changes not yet implemented in routine care, one involved an eHealth tool to facilitate data collection and teleconsultations [37, 45]. Another change addressed comorbidities (i.e., GERD) by collaborating with experts [39]. Furthermore, one change described a support and advocacy group for patients with IPF and three changes aimed to empower patients in coping with IPF by organizing group sessions consisting of information provision and coping or mindfulness strategies [39, 40, 42]. Lastly, one change focused on patients’ palliative care needs by proposing a collaboration with experts from the community setting [31].

### Which elements of the Chronic Care Model did the changes target?

The 13 changes targeted a median of two elements of the CCM (range 1–5) and none targeted all six elements of the CCM (see Table 3 CCM elements). The delivery system design (n = 10, 77%) and self-management support (n = 9, 69%) were used the most, followed by decision support (n = 5, 38%). Clinical information system and community linkages were addressed in four care changes, respectively (31%). We classified two changes (15%) under healthcare organization. The change described by Bajwah and colleagues and the one described by Sharp and Barrat and their colleagues targeted the most elements (5/6) [31, 33, 34]. Three initiatives targeted only one CCM element [36, 42, 46]. The next paragraphs describe the building blocks that were targeted in more detail (see Table 1 for a definition).

**Table 3:** Overview of the Chronic Care Model elements

	Elements of the Chronic Care Model (CCM)						Number of CCM components targeted
	Healthcare organization	Delivery system design	Self-management support	Clinical information system	Decision support	Community linkages	
Hospital2Home [31]		X	X	X	X	X	5
MDT approach [39]		X			X		2
PRISIM [46]			X				1
Support group [40]			X			X	2
IPF-Online [37, 45]		X	X	X			3
MBSR program [36]			X				1
PPEPP [32]		X	X				2
SCDAT and MDT-meeting [33, 34]	X	X		X	X	X	5
NPP [41]		X	X				2
IPF care [43]		X	X				2
Educational initiative [35]	X	X		X	X		4
Care coordinator [42]		X					1
MDC Care Model [38, 44]		X	X		X	X	4
Numbers of changes that targeted the CCM component	2 (15%)	10 (77%)	9 (69%)	4 (31%)	5 (38%)	4 (31%)	

*MDT* multidisciplinary team, *PRISIM* program to reduce idiopathic pulmonary fibrosis symptoms and improve management, *PPEPP* patient and partner empowerment program, *MBSR* mindfulness-based stress reduction program, *SCDAT* supportive care decision aid tool, *MDC* multidisciplinary collaborative, *NPP* named patient program

#### Delivery system design

Four changes comprised multidisciplinary teams with distinct roles, including experts on GERD as well as community care providers or members of community services [31, 33, 38, 39, 44]. Also, a care coordinator was included in routine care and the role of a nurse specialized in PF/ILD care was highlighted in a nurse-led telephone program and a pharmacological management program [41–43]. Two changes also actively involved patients and caregivers to obtain shared decision making regarding their care [31, 38]. To involve different team members, several forms were used, including case conferences, collaborative care models or team meetings [31, 34, 38, 39]. Team members had a role in providing additional expertise or in delivering self-management support such as patient education. The changes used regular follow-up visits either via face-to-face meetings, telephone contacts or virtual consultations.

#### Self-management support

Self-management support included education on IPF and its treatment, coping strategies, strategies to overcome breathlessness, breathing techniques, or referral to community services [32, 36–38, 40, 41, 43, 45, 46]. Two changes included patient involvement in their care decisions, using individual care plans to inform care based on patients’ preferences and goals [31, 38]. Formats to provide self-management support included group sessions, nurse-led telephone support or home visits, multidisciplinary ILD collaborative care, nurse-led case conferences, mindfulness-based stress reduction programs or using an eHealth tool [31, 32, 36, 36–38, 43, 45, 46].

#### Clinical information system

Four changes used data to optimize patient care. One change gathered data on the performance of providers, used data to send out reminders and developed order sets in the electronic medical record to support providers in the delivery of care [35]. A second change included a supportive care decision aid tool aiming to prompt care providers to assess and identify patients requiring discussions on palliative and supportive care needs or referral [34]. Two changes also used the clinical information system to share patient information and care decisions among team members [31, 33]. An eHealth tool collected patient data, including lung function tests and results of patient-reported outcomes, and used patient data to generate e-mail alerts to care providers in case patients needed additional support [37, 45].

#### Decision support

Five changes targeted decision support. Bajwah and colleagues developed and used a new guideline on symptom control [31]. Another change provided additional education and training to care providers on the content and processes of care delivery [35]. Also, several changes integrated the expertise of additional specialists to attain care decisions during multidisciplinary meetings, as part of a multidisciplinary care model or during case conferences [31, 33, 38, 39].

#### Community linkages

Collaboration with community services regarding palliative care and supportive needs were mentioned in two changes for which a collaborative model or a case conference were used [31, 38]. Also, referrals to community services were reported and one initiative reported the role of a patient advocacy group [33, 40].

#### Health organization

Two changes involved a quality improvement process to assess and optimize the care delivery processes [34, 35]. One research team used key performance metrics, thereby identifying issues in their local practice patterns and informing improvement strategies [35]. Other researchers based the content of their change on an assessment of patients’ unmet needs and practice patterns [34].

### Which type of outcome did the changes assess?

Of the seven changes not yet implemented in routine care, six focused on patient-reported outcomes (PROs). Feasibility, safety and/or acceptability outcomes were assessed in four changes and clinical outcomes in one. Four changes also assessed the experiences and satisfaction of stakeholders regarding the change.

Of the six changes already implemented in routine care, one provided a description of care only, without mentioning outcomes or evaluation. Of the other five, all evaluated stakeholders’ experiences and satisfaction with the changes, two reported process measures, one used PROs, two looked at the impact on healthcare utilization and one evaluated feasibility outcomes.

Zooming in on the patient outcomes, five reported health-related quality of life (HRQoL), followed by anxiety and depression (n = 4), perceived stress (n = 3), symptoms (n = 3), preferred place of death (n = 2), reported palliative care needs (n = 1), emotional well-being (n = 1) and/or mood (n = 1). Informal caregivers also received questionnaires to assess their quality of life (n = 3), anxiety and/or depression (n = 2), well-being (n = 1), perceived stress (n = 1) and/or care burden (n = 1).

## Discussion

Growing evidence on the burden of IPF disease resulted in efforts to offer timely and comprehensive patient-centred care throughout the disease trajectory [47]. Our systematic review identified 13 changes that focused on redesigning care models for IPF patients or components thereof.

Changes focused mainly on advanced care planning/palliative care, supporting patients in living with IPF and on the overall organisation of a care program. The most commonly addressed CCM components were delivery system design and self-management support. Delivery system design implies how care is delivered, identifying changes towards team-based or multidisciplinary care. The role of a nurse specialized in IPF/ILD was frequently cited. Self-management support mainly encompassed providing education, but other support strategies to help patients dealing with IPF and its consequences were less mentioned [48]. Linkages between outpatient clinics and community care were reported infrequently in the studies included. This is consistent with findings in other disease populations, but in contrast to our review, others found changes in the clinical information system to be reported the most based on an analysis of changes in care in 42 heart failure, depression, diabetes and asthma programs [49]. We furthermore showed that practice redesign almost always targeted multiple CCM components. This is not surprising as CCM elements are intertwined and evidence did not yet identify one single essential element to target when aiming to improve outcomes [29, 49]. We also found a high variability in changes reported. This variability is observed in studies on other diseases and it challenges the identification of a core set of elements that might lead to improvements in clinical care [50].

A meta-analysis in four other chronic illnesses showed that interventions targeting CCM elements improved clinical outcomes and processes but had less impact on health-related quality of life [29]. In our review, patient outcomes were targeted the most, particularly health-related quality of life. Processes were assessed in only two changes. In IPF care, a recent review highlighted the potential of PROs and experiences to improve the patient-centredness of the care delivery pathway [51]. However, no core outcome set is available yet for IPF research and care. This is a crucial gap if one wants to evaluate whether changes in care spin off in better clinical and patient-reported outcomes or care processes [52].

Recent reviews highlighted the importance of pharmacological management of IPF, palliative care, the potential use of PROs and the multidimensional supportive needs of patients and caregivers [11, 51, 53–55]. Our review adds to this by evaluating care models and components thereof from the perspective of chronic integrated care.

The quality analysis of the included studies revealed that there were several methodological shortcomings, mainly at the level of reporting. More specifically, we showed a lack of information on how the intervention was developed and to what extent the intervention was delivered as intended. Overall, evidence is scarce and heterogeneous, and we would like to call researchers to develop, evaluate, implement, and report care models, including its complex interventions in a thorough and transparent way so that research, and ultimately clinical care can move forward. We outline some factors to consider when developing and/or implementing changes in care.

Most articles only briefly described the development of their change by for instance using frameworks and quality improvement methods. However, five of the 13 identified changes did not describe the development phase, and none mentioned the use of the CCM explicitly. Yet, several CCM toolkits are available to guide future intervention development and implementation. The Medical Research Council (MRC) guide on complex interventions, in which stakeholder involvement is stressed, might also be a relevant resource [56]. Of note, only 46% (n = 6) of our identified changes involved stakeholders in their development phase, of which only two involved patients. A close collaboration with stakeholders is increasingly being put forward as a key ingredient to make the envisioned changes more acceptable and useful, and enhance the potential of a successful and sustainable implementation [57, 58]. Because of the high variability in settings and contexts in which changes are envisioned, a detailed context analysis and description is crucial in view of replicability or scalability of findings to other settings [58]. Of the six changes in our review that were already implemented in routine care, only two clearly mentioned an analysis of their context as part of their quality improvement process. Potential barriers and facilitators should be addressed as both play an enormous role when aiming to redesigning care [59]. For instance, one of the included papers discussed several barriers and facilitators, including the need for resources, training and the availability of a clinical information system [35]. Work outside IPF stresses the importance of having a team ready and motivated to attain change as well as the involvement of leadership in order to be successful in improving care [59].

### Limitations

We only included articles that particularly mentioned a change in the content or process of a care program, hereby excluding articles that made recommendations only. We did not include grey literature, nor did we survey IPF programs on unpublished improvements in care, which, despite our rigorous search strategy, could have led to missing out relevant changes. Moreover, given that none of the included articles used the CCM elements, classification errors in describing the content of the changes are possible, although two researchers made the classification independently. Lastly, we could only rely on the information provided in the articles. It is thus possible that crucial information was missed, which again highlights the importance of transparency and completeness of reporting.

## Conclusions and implications

This systematic review describes the characteristics of 13 new care models/components for IPF, using the CCM elements. Evidence is scarce and heterogeneous. Most changes focused on care delivery and self-management support. There is a need to develop, evaluate and implement new models of care for IPF and to comprehensively describe the particular setting and context in order to be able to move clinical care for patients with IPF forward.

## Supplementary Information

**Additional file 1. **PRISMA checklist 2009.

**Additional file 2.** Final search string in PubMed/Medline.

**Additional file 3.** Overview of study characteristics.

**Additional file 4.** Overview of the research phases.

**Additional file 5.** Quality assessment of included articles according to the Mixed Methods Appraisal Tool.

**Additional file 6.** Overview of the characteristics of the identified care models and components.

## Data Availability

All the data (e.g., data retrieved from the included manuscripts, search string in Medline/PubMed) are included in this article and its Additional files.

## Abbreviations

CCM: Chronic care model
CRD: Centre for reviews and dissemination
DMAIC: Define measure analyze improve compare
GERD: Gastro-esophageal reflux disease
HRQoL: Health-related quality of life
IPF: Idiopathic pulmonary fibrosis
MMAT: Mixed methods appraisal tool
MRC: Medical Research Council
PDCA: Plan, Do, Study, Act cycles
PF-ILDs: Progressive fibrosing interstitial lung diseases
PRO: Patient-reported outcome
WHO: World Health Organization
