# Supplementary material for: Care programs and their components for patients with idiopathic pulmonary fibrosis: a systematic review
Source: Respir Res. 2021 Aug 16;22:229. doi: 10.1186/s12931-021-01815-8 (PMC8365984; doi:10.1186/s12931-021-01815-8)
Supplement: Supplementary file 2 — Additional file 2. Final search string in PubMed/Medline. [file 12931_2021_1815_MOESM2_ESM.docx]

Additional file 2: Final search string in PubMed/Medline

((((((((((((Lung Diseases, Interstitial[Mesh]) OR interstitial-pneumopath* [Tiab] OR interstitial-lung-dis* [Tiab]) OR parenchymal-lung-disease* [Tiab]) OR DPLD [Tiab]) OR ILD [Tiab]) OR interstitial-pneumoni*[Tiab]) OR fibrosing-alveolit* [Tiab]) OR idiopathic-pulmonary-fibros* [Tiab]) OR IPF [Tiab])))) AND ((((((((((((((((((((((((((((((((((((((((((((((((((((((((((((("Patient Care"[Mesh]) OR patient care [Tiab]) OR informal-care* [Tiab]) OR long-term-care [Tiab]) OR chronic-care [Tiab]) OR illness-management [Tiab]) OR disease-management [Tiab]) OR care-continu* [Tiab]) OR nursing-care [Tiab]) OR holistic-nursing [Tiab]) OR palliative-care [Tiab]) OR palliative-treatment [Tiab]) OR palliative-therap* [Tiab]) OR terminal-care [Tiab]) OR care-team* [Tiab]) OR interdisciplinary-health-team* [Tiab]) OR multidisciplinary-care [Tiab]) OR collaborative-care [Tiab]) OR multidisciplinary-team*[Tiab]) OR care-goal* [Tiab]) OR advance-care-planning [Tiab]) OR case-management [Tiab]) OR critical-path [Tiab]) OR care-planning[Tiab]) OR critical-pathway* [Tiab]) OR clinical-pathway* [Tiab]) OR clinical-path [Tiab]) OR Patient Care Management[Mesh]) OR patient-care-management [Tiab]) OR comprehensive-care [Tiab]) OR practice-pattern* [Tiab]) OR donabedian-model [Tiab]) OR outcome-assessment* [Tiab]) OR process-assessment* [Tiab]) OR outcome-measure [Tiab]) OR process-measure [Tiab]) OR Self-Management[Mesh]) OR Self Care[Mesh]) OR "self care" [Tiab]) OR "self management" [Tiab]) OR health-care-quality [Tiab]) OR patient-centered-care [Tiab]) OR patient-centred-care [Tiab]) OR patient-focused-care [Tiab]) OR end-of-life[Tiab]) OR quality-improvement*[Tiab]) OR Holistic Health[Mesh]) OR holistic-health [Tiab]) OR holistic-therap* [Tiab]) OR holistic-care [Tiab]) OR holistic-medicine [Tiab]) OR Patient Education as Topic[Mesh]) OR patient-education [Tiab]) OR chronic-treatment*[Tiab]) OR ambulatory-monitoring[Tiab]) OR health-care-evaluation[Tiab]) OR nursing-practice[Tiab]) OR outcome-management[Tiab]) OR long-term-therap*[Tiab]) OR outpatient-care [Tiab]) OR ambulatory-care [Tiab])
