# Supplementary material for: Care programs and their components for patients with idiopathic pulmonary fibrosis: a systematic review
Source: Respir Res. 2021 Aug 16;22:229. doi: 10.1186/s12931-021-01815-8 (PMC8365984; doi:10.1186/s12931-021-01815-8)
Supplement: Supplementary file 3 — Additional file 3. Overview of study characteristics. [file 12931_2021_1815_MOESM3_ESM.docx]

Additional file 3. Overview of study characteristics

|  | Aim of the study | Design (as reported in the articles) | Study population and characteristics | Measurement points of main outcomes |
| --- | --- | --- | --- | --- |
| Articles describing a care program or component thereof without implementation in routine clinical care yet | | | | |
| Bajwah 2015  The United Kingdom | *“[…] to obtain preliminary information in what ways Hospital2Home influences the palliative care concerns of patients with advanced fibrotic interstitial lung disease (ILD) and their carers, and to evaluate the feasibility and acceptability of the intervention in this group.”* | A fast-track randomized controlled trial with embedded qualitative interview | Included patients (baseline data): n=53  -IPF: n=44; NSIP: n=9  Merged baseline participants’ characteristics  -Mean age (SD): 68,9 (10,6)  -Gender: 72% male  -Mean DLco predicted (SD): 24 (8,5) | Timepoints IG of primary outcome:  -Baseline, 4 weeks and 8 weeks after intervention  Timepoints CG of primary outcome:  -Baseline, before intervention, 4 weeks after intervention  Qualitative interviews conducted after completion study  Feasibility criteria after completion study |
| Jones 2018  The United Kingdom | *“[…] to investigate whether pH-impedance and BALF studies could be combined to assess reflux and aspiration in IPF. We developed a novel ‘aerodigestive’ multidisciplinary team (MDT) approach to support personalized treatment in clinical practice.”* | Prospective observational study | *Remark: Data of patients who completed the study*  Characteristics IPF patients n= 36  -Median age: 73 years  -Gender: 75% male  -Median FEV_1_ predicted (range): 83,5% (46,8-163,6)  -Median vital capacity predicted (range): 77,6% (47,9-146,6) | Cross-sectional at time of evaluation |
| Lindell 2010  The United States | *“[…] to test the ability of the Program to Reduce Idiopathic Pulmonary Fibrosis Symptoms and Improve Management (PRISIM) to decrease symptom burden, decrease stress, and improve perceptions of HRQoL for patients with IPF and their care partners.”* | Quantitatively driven, concurrent nested mixed-method design: Randomized trial | Enrolment of 42 participants:  -IPF patients: n=21; Care partners: n=20  Merged data of patients’ characteristics:  -Mean age (SD): 66,2 years (10,9)  -Gender: 76% male  -FVC (n=20)  -FVC > 55% predicted: n=14  -FVC 50-55% predicted: n=3  -FVC <50% predicted: n=3 | IG:  -PROs: Before and after intervention  -Interviews after intervention |
| Magnani 2017  Italy | *“[…] to evaluate the benefits of a support group led by a nurse, for patients affected by IPF and their family members, to improve the psychological well-being, in its six dimensions: vitality, self-control, positivity, general health, depressed mood and anxiety. “* | A single group pre-post study | Enrolment of 18 participants:  -IPF patients: n=10; Relatives: n=8  Characteristics of participants: n=18  -Mean age (SD): 66,5 years  -Gender: 55% women | PRO: baseline (before) and after 6 months (after attending group) |
| Moor 2018  The Netherlands | *“Together with patients we developed ‘IPF-online’, an e-health tool for patients with IPF, and to evaluate its feasibility and the user satisfaction of this tool.”* | NR | Inclusion of 27 IPF patients    Baseline characteristics patients  -Mean age (range): 67 years (56-86)  -Gender: 85% male  -Median FVC predicted (range): 78% (46-131)  -Mean DLco predicted (range): 50% (16-79%) | PROs and medication use: baseline and after 14 days in eHealth tool  Patient experiences: after completion study |
| Moor 2018 (respir)  The Netherlands | To assess the feasibility of ‘IPF-online’, integrated with real-time wireless home spirometry and to evaluate potential barriers and solutions for implementation of wireless home spirometry. | A prospective pilot study | Inclusion of 10 IPF patients  Baseline characteristics patients  -Mean age: 71 years  -Gender: n=9 male (75%)  -Mean FVC predicted: 79% | PROs: before and after 4 weeks in eHealth tool  Daily home spirometry  Patient experiences: after completion study |
| Sgalla 2015  Italy | To study the safety and the feasibility of a Mindfulness-Based Stress Reduction (MBSR) program and to assess its efficacy on mood, quality of life, and pulmonary function. | A prospective observational pilot study | Inclusion of 19 patients  -IPF: n=12; NSIP: n=2; RA-ILD: n=1; Chronic HP: n=1; CTD-ILD: n=1; Asbestosis: n=1; DIPNECH: n=1  Baseline characteristics patients: n=19  -Mean age (SD): 65 years (8)  -Gender: 11 men, 8 women  -Mean FVC predicted (SD): 82% (26)  -Mean DLco predicted (SD): 51% (19) | Primary outcome: safety measured after completion study |
| Van Manen 2017  The Netherlands | To assess the effect of a short multidisciplinary patient and partner empowerment programme (PPEPP) on the quality of life of patients with IPF and their relatives. | NR | Inclusion of 46 participants  Included in data analysis: 40 participants  Patients’ characteristics in the IG: n=13  -Median age (range): 63 years (54-74)  -Gender: 77% men, n=10  -Median FVC predicted (range): 80% (50-100)  -Median DLco predicted (range): 46% (25-60) | PROs: Baseline, after 3 weeks and after 3 months  Patient experiences: after completion study |
| Articles describing a care program or component thereof which is implemented in routine care | | | |  |
| Barrat 2018  The United Kingdom | To assess the effectiveness of a novel collaborative multidisciplinary team (MDT) meeting on the patient’s assessment of palliative care needs. | Pre-post study | Pre-MDT group n=26  Post MDT n=46  Merged data baseline characteristics patients  -Mean age (SD): 71,1 years (2,4)  -Gender: 71,2% male  -Mean FVC predicted (SD), n=70: 58,9% (2,3)  -Mean DLco predicted (SD), n=43: 31,2% (3) | Pre-cohort: records of patients who passed away 8 months prior to MDT introduction  Post-cohort: patient records between January 2016 and July 2016 |
| Chaudhuri 2014  The United Kingdom | To demonstrate the authors’ experiences in prescribing Pirfenidone in a named patient program. | Observational retrospective study | 40 IPF patients included  Baseline characteristics patients: n=40  -Mean age (range): 65,8 years (48-80)  -Gender: 70% male  -Mean FVC (range): 77,3% (46-146)  -Mean DLco (range): 42,4% (14-81) | 3, 6 and 9 months from start pirfenidone |
| Duck 2017  The United Kingdom | To describe two IPF Care Patient Support Programs in the UK and in Austria, aiming to support adherence to medication taking; and to describe its benefits to patients | NR | Program UK:  -May 2013-October 2014: n=465 IPF patients enrolled  Program Austria:  -November 2013-till time of writing of article: n=69 IPF patients enrolled | Austria: 8-month period  UK= 18-month period |
| Fernandez Perez 2018  The United States | To design and implement a project aimed at measuring key quality indicators and how they may impact clinical practice and IPF patient perception of care. | Quality improvement study | Baseline in 2014: n=267 IPF patients  Evaluation: prospective cohort in 2016-2017: n=587 IPF patients  Process evaluation:  -Survey: 50 patients and ILD team members  -Focus group: patients and nurses | Metrics: quarterly measures between 2016 and 2017  Comparison with year 2014 as baseline  Stakeholder experiences after completion study |
| Hambly 2019  Canada | *“[…] to evaluate the impact of a coordinator on IPF patient satisfaction and HRQoL.”*  *“[…] to assess the economic impact of including a coordinator in the management of IPF patients.”* | Cross-sectional study | Enrolment of 40 IPF patients  -High coordinator use (HCU): n=20  -Low coordinator use (LCU): n=20  Merged data baseline characteristics patients  -Mean age (SD): 73,7 years (6)  -Gender: 83% men  -Mean FVC predicted (SD): 69,9% (17,7)  -Mean DLco predicted (SD): 39,7% (12,8) | PROs: cross-sectional |
| Kalluri (1) 2014  Canada | To report about their approach to the assessment and management of dyspnea using a collaborative, rehabilitative and palliative approach. | Case report | One case report: patient with familial IPF  Characteristics patient:  -Age: 59 years  -Gender: male | NA |
| Kalluri (2) 2017  Canada | *“[…] to describe a case where patient-centred multidisciplinary care provided early integrated palliative care and supported a very active end-of-life experience for a patient with IPF, including dying at home […].”* | Case report | One case report: patient with IPF  Characteristics patient:  -Age: 72 years  -Gender: male | NA |
| Kalluri 2018  Canada | To review consecutive IPF death in their ILD clinic and to compare acute care utilization and hospital deaths before and after institution of their new Multidisciplinary Collaborative (MDC) care model. | Pre-post retrospective study | IG (=patients receiving MDC care): n=22  CG (=patients who died before MDC care was implemented): n=10  Merged data baseline characteristics  -Mean age (SD): 70,8 years (12,3)  -Gender: 62% male  -Mean FVC predicted (SD): 66,7% (14,4)  -Mean DLco predicted (SD): 41,6% (15,8) | Healthcare utilization: period between first visit and time of death  IG: patient records between 2012-2016  CG: patient records between 2009-2012 |
| Pooler 2018  Canada | *“[…] to explore bereaved caregivers’ experiences and perception of an early integrated palliative approach implemented at a Multidisciplinary Interstitial Lung Disease Clinic.”* | Qualitative study | Characteristics caregivers: n=8  -Gender: 7 female, 1 male  -4 adult children, 4 spouses | Interviews in 2016 after passing away of the patient |
| Sharp 2018  The United Kingdom | To investigate current practices around palliative and supportive care and, to develop a supportive care decision aid tool, and to explore its impact. | Quality improvement study | Three cohorts:  CG1: pre-tool: n=89  CG2: deceased patients: n=64  IG: after-tool: n=73  Merged characteristics  -Mean age (SD): 74,8 years (8,4)  -Gender: 80,6% male  -Mean FVC predicted (SD): 77% (20,6)  -Mean DLco predicted (SD): 44,5% (16,4) | Cross-sectional before tool (=CG1) and after introduction tool (=IG) |

Legend with abbreviations: **NR** (not reported), **IG (**intervention group), **CG** (control group), **ILD** (interstitial lung disease), **POS** (palliative care outcome scale), **IPF** (idiopathic pulmonary fibrosis), **NSIP** (nonspecific interstitial pneumonia), **SD** (standard deviation), **FVC** (forced vital capacity), **DLco** (diffusion capacity for carbon monoxide), **BALF** (bronchoalveolar lavage fluid), **MDT** (multidisciplinary team), **FEV_1_** (forced expiratory volume in 1 sec), **PRISIM** (Pulmonary Fibrosis Symptoms and Improve Management), **HRQoL** (health-related quality of life), **MBRS** (Mindfulness-Based Stress Reduction), **RA-ILD** (rheumatoid arthritis-associated interstitial lung disease), **HP (**hypersensitivity pneumonitis), **CTD-ILD** (connective tissue disease-associated interstitial lung disease), **DIPNECH** (Diffuse idiopathic pulmonary neuroendocrine cell hyperplasia), **PPEPP** (patient and partner empowerment programme), **CPFE** (combined pulmonary fibrosis and emphysema), **NSIP** (Non-specific interstitial pneumonia), **COP** (cryptogenic organizing pneumonia), **HCU** (high coordinator use), **LCU** (low coordinator use), **MD**C (multidisciplinary collaborative care)

References not cited in the main text

1. Kalluri M, Richman-Eisenstat J. Breathing is not an option; dyspnea is. J Palliat Care. 2014; 30(3):188–91.
2. Kalluri M, Richman-Eisenstat J. Early and Integrated Palliative Care to Achieve a Home Death in Idiopathic Pulmonary Fibrosis. J Pain Symptom Manage. 2017; 53(6):1111–5.
