# Supplementary material for: Care programs and their components for patients with idiopathic pulmonary fibrosis: a systematic review
Source: Respir Res. 2021 Aug 16;22:229. doi: 10.1186/s12931-021-01815-8 (PMC8365984; doi:10.1186/s12931-021-01815-8)
Supplement: Supplementary file 4 — Additional file 4. Overview of the research phases. [file 12931_2021_1815_MOESM4_ESM.docx]

Additional file 4. Overview of the research phases

|  |  |  | Main focus of the article | | | |
| --- | --- | --- | --- | --- | --- | --- |
|  |  | Name of the change | Description of the change* | Feasibility/piloting of the change | Evaluation of the change | Quality improvement |
| Overview of the care programs or interventions, without implementation in routine clinical care yet (n=7) | Bajwah 2015 | Hospital2Home |  | X |  |  |
|  | Jones 2018 | MDT approach |  | X |  |  |
|  | Lindell 2010 | PRISIM |  |  | X |  |
|  | Magnani 2017 | Support group |  | X |  |  |
|  | Moor 2018, | IPF-Online |  | X |  |  |
|  | Moor 2018 (respir re) |  |  | X |  |  |
|  | Sgalla 2015 | MBSR program |  | X |  |  |
|  | Van Manen 2017 | PPEPP |  | X |  |  |
| Overview of the care programs or interventions, implemented in routine care (n=6) | Sharp 2018 | SCDAT and MDT-meeting |  |  |  | X |
|  | Barrat 2018 |  |  |  | X |  |
|  | Chaudhuri 2014 | NPP | X |  |  |  |
|  | Duck 2017 | IPF care |  |  | X |  |
|  | Fernandez-Perez 2018 | Educational initiative |  |  |  | X |
|  | Hambly 2019 | Care coordinator |  |  | X |  |
|  | Kalluri 2014 | MDC Care Model | X |  |  |  |
|  | Kalluri 2017 |  | X |  |  |  |
|  | Kalluri 2018 |  |  |  | X |  |
|  | Pooler 2018 |  |  |  | X |  |

Legend with abbreviations: **MDT** (multidisciplinary team), **PRISIM** (program to reduce idiopathic pulmonary fibrosis symptoms and improve management), **PPEPP** (patient and partner empowerment program), **MBSR** (mindfulness-based stress reduction program), **SCDAT** (supportive care decision aid tool), **MDC** (multidisciplinary collaborative), **NPP** (named patient program)

*(only a description of the change was given in the article, without feasibility or evaluation of the change)
