# Supplementary material for: Care programs and their components for patients with idiopathic pulmonary fibrosis: a systematic review
Source: Respir Res. 2021 Aug 16;22:229. doi: 10.1186/s12931-021-01815-8 (PMC8365984; doi:10.1186/s12931-021-01815-8)
Supplement: Supplementary file 5 — Additional file 5. Quality assessment of included articles according to the Mixed Methods Appraisal Tool. [file 12931_2021_1815_MOESM5_ESM.docx]

Additional file 5. Quality assessment of included articles according to the Mixed Methods Appraisal Tool (MMAT)

|  |  | Bajwah 2015 | Jones 2018 | Lindell 2010 | Magnani 2017 | Moor 2018 | Moor 2018 (respir) | Sgalla 2015 | Van Manen 2017 | Barrat 2018 | Chaudhuri 2014 | Duck 2017 | Fernandez -perez 2018 | Hambly 2019 | Kalluri 2018 | Pooler 2018 | Sharp 2018 |
| --- | --- | --- | --- | --- | --- | --- | --- | --- | --- | --- | --- | --- | --- | --- | --- | --- | --- |
| Screening questions | S1. Are there clear research questions? | Yes | Yes | Yes | Yes | Yes | Yes | Yes | Yes | Yes | No | Yes | Yes | Yes | Yes | Yes | Yes |
|  | S2. Do the collected data allow to address the research questions? | Yes | Yes | Yes | Yes | Yes | Yes | Yes | Yes | Yes | No | Yes | Yes | Yes | Yes | Yes | Yes |
| 1.  Qualitative design | 1.1. Is the qualitative approach appropriate to answer the research question? | Yes |  | Yes |  |  |  |  |  |  |  |  | Yes |  |  | Yes |  |
|  | 1.2. Are the qualitative data collection methods adequate to address the research question? | Yes |  | Yes |  |  |  |  |  |  |  |  | Can’t tell |  |  | Yes |  |
|  | 1.3. Are the findings adequately derived from the data? | Yes |  | Can’t tell |  |  |  |  |  |  |  |  | Can’t tell |  |  | Yes |  |
|  | 1.4. Is the interpretation of results sufficiently substantiated by data? | Yes |  | Can’t tell |  |  |  |  |  |  |  |  | No |  |  | Yes |  |
|  | 1.5. Is there coherence between qualitative data sources, collection, analysis and interpretation? | Yes |  | Can’t tell |  |  |  |  |  |  |  |  | Can’t tell |  |  | Yes |  |
| 2. Quantitative randomized controlled trials | 2.1. Is randomization appropriately performed? | Yes |  | Yes |  |  |  |  |  |  |  |  |  |  |  |  |  |
|  | 2.2. Are the groups comparable at baseline? | Yes |  | Yes |  |  |  |  |  |  |  |  |  |  |  |  |  |
|  | 2.3. Are there complete outcome data? | Yes |  | Yes |  |  |  |  |  |  |  |  |  |  |  |  |  |
|  | 2.4. Are outcome assessors blinded to the intervention provided? | Can’t’ tell |  | Can’t tell |  |  |  |  |  |  |  |  |  |  |  |  |  |
|  | 2.5 Did the participants adhere to the assigned intervention? | Yes |  | Can’t tell |  |  |  |  |  |  |  |  |  |  |  |  |  |
| 3. Quantitative nonrandomized | 3.1. Are the participants representative of the target population? |  |  |  | Can’t tell |  |  | Can’t tell | Yes | Yes |  |  | Can’t tell |  | Yes |  | Can’t tell |
|  | 3.2. Are measurements appropriate regarding both the outcome and intervention (or exposure)? |  |  |  | Yes |  |  | Yes | Yes | Yes |  |  | Yes |  | Yes |  | Yes |
|  | 3.3. Are there complete outcome data? |  |  |  | Yes |  |  | No | Yes | Can’t tell |  |  | Can’t tell |  | Yes |  | Yes |
|  | 3.4. Are the confounders accounted for in the design and analysis? |  |  |  | Can’t tell |  |  | Can’t tell | No | Can’t tell |  |  | Can’t tell |  | No |  | Can’t tell |
|  | 3.5. During the study period, is the intervention administered (or exposure occurred) as intended? |  |  |  | Can’t tell |  |  | Can’t tell | Can’t tell | Can’t tell |  |  | Can’t tell |  | Can’t tell |  | Can’t tell |
| 4.Quantitative descriptive | 4.1. Is the sampling strategy relevant to address the research question? |  | Yes |  |  | Yes | Yes |  |  |  | Yes | Yes |  | Can’t tell |  |  |  |
|  | 4.2. Is the sample representative of the target population? |  | Can’t tell |  |  | Can’t tell | Can’t tell |  |  |  | Yes | Can’t tell |  | Can’t tell |  |  |  |
|  | 4.3. Are the measurements appropriate? |  | Yes |  |  | Yes | Yes |  |  |  | Yes | Yes |  | Yes |  |  |  |
|  | 4.4. Is the risk of nonresponse bias low? |  | No |  |  | Can’t tell | Yes |  |  |  | Can’t tell | Can’t tell |  | Yes |  |  |  |
|  | 4.5. Is the statistical analysis appropriate to answer the research question? |  | Yes |  |  | Can’t tell | Yes |  |  |  | Can’t tell | Can’t tell |  | Yes |  |  |  |
| 5. Mixed methods | 5.1. Is there an adequate rationale for using a mixed method design to address the research question? | Yes |  | Yes |  |  |  |  |  |  |  |  | Yes |  |  |  |  |
|  | 5.2. Are the different components of the study effectively integrated to answer the research question? | Yes |  | Yes |  |  |  |  |  |  |  |  | No |  |  |  |  |
|  | 5.3. Are the outputs of the integration of qualitative and quantitative components adequately interpreted? | Yes |  | Yes |  |  |  |  |  |  |  |  | Can’t tell |  |  |  |  |
|  | 5.4. Are divergences and inconsistencies between quantitative and qualitative results adequately addressed? | Yes |  | Yes |  |  |  |  |  |  |  |  | Can’t tell |  |  |  |  |
|  | 5.5. Do the different components of the study adhere to the quality criteria of each tradition of the methods involved? | Yes |  | Can’t tell |  |  |  |  |  |  |  |  | No |  |  |  |  |

Note: Only the criteria corresponding the design of the article in question need to be scored. In case of a mixed methods design, both the qualitative and quantitative design must be scored additionally.

Note: The research team decided to use 80% as a complete outcome data value.

Note: Answer options ‘yes’ (green), ‘no’ (red) and ‘can’t tell’ (orange).
