# Supplementary material for: Care programs and their components for patients with idiopathic pulmonary fibrosis: a systematic review
Source: Respir Res. 2021 Aug 16;22:229. doi: 10.1186/s12931-021-01815-8 (PMC8365984; doi:10.1186/s12931-021-01815-8)
Supplement: Supplementary file 6 — Additional file 6. Overview of the characteristics of the identified care models and components. [file 12931_2021_1815_MOESM6_ESM.docx]

Additional file 6: Overview of the characteristics of the identified care models and components

| Name of the program  First author and date | What is the goal or hypothesis of the program? | How was the program developed? | What is the conclusion of the paper regarding the program? |
| --- | --- | --- | --- |
| Overview of the care program or component thereof without implementation in routine clinical care yet (n=7) | | | |
| Hospital2Home  Bajwah 2015 (31) | *“[…] to provide a quality comprehensive palliative care assessment and streamlining of transfer of data between specialist and community settings improving coordination of care and communication […]”* | Intervention developed using Medical Research Council guidance and informed by previous work including a systematic review and qualitative interviews. | The intervention was considered feasible and accepted.  A positive effect was observed on the palliative concerns, quality of life, anxiety and depression.  The intervention made discussions on advanced care planning possible. |
| Aerodigestive multidisciplinary team  Jones 2018  (39) | To better detect gastro-oesophageal reflux disease (GORD) and treat it, as GORD is a known risk factor for poor outcome in patients with IPF | NR | The multidisciplinary team was able to make recommendations for medical therapy, referral for additional investigations and referral to anti-reflux surgery.  Demonstration of the safety and feasibility of an integrated assessment of gastro-oesophageal reflux and aspiration. |
| PRISIM  (Program to reduce IPF symptoms and improve management)  Lindell 2010  (46) | Participation would improve perceptions of HRQoL and decrease the burden of symptoms. | Content developed by a pulmonary clinical nurse specialist, a psychiatric clinical specialist (training as a cognitive behavioural therapist) and an advanced care planning instructor. | Quantitative data showed a negative effect on anxiety and physical QoL, but qualitative data suggested a positive satisfaction on the participation and positive benefits (e.g. less isolation). |
| Nurse-led support group  Magnani 2017  (40) | To set up a network, share experiences and receive/give comfort, and work on awareness-raising initiatives | NR | The findings show an increase in the psychological well-being. |
| IPF online  Moor 2018  (37,45) | To collect data at home and facilitate consultations at distance | Based on patients’ wish to ‘track’ their disease’  Based on evidence from literature, experiences in other fields and patient suggestions. | IPF-online was considered feasible and accepted by patients.  Also, the results showed no negative impact on anxiety levels and a stable quality of life (as shown with the K-BILD total score) |
| MBSR (Mindfulness-based stress reduction program)  Sgalla 2015  (36) | Participation would have an impact on mood, quality, and pulmonary function (practice might indirectly trigger a positive, measurable effect on pulmonary performance and exercise tolerance) | MBSR training program created in 1979 by J Kabat-Zinn. Now a standardised program used in other disorders. | MBSR was considered feasible and safe. A positive effect was seen on patients’ moods. |
| PPEPP  (Patient and Partner Empowerment Programme for IPF)  Van Manen 2017  (32) | To improve the quality of life (wellbeing) of patients and their partners. | Theoretical basis: NR, however the content of some sessions was based on theoretical models, such as a stress-coping model.  Involvement of a pulmonologist, a nurse specialized in ILD, an oxygen supplier, a social worker, physiotherapists, two IPF patients and a vicar. | PPEPP was accepted by patients and partners.  The programme showed an improvement in QoL. |
| Overview of the care programs or components thereof with implemented in routine care (n=6) | | | |
| SCDAT (Supportive care decision aid tool)  Collaborative multidisciplinary team meeting  Sharp 2018  Barrat 2018  (33,34) | To support clinicians in identifying patients with potential palliative and supportive care needs. To improve documentation of end-of-life discussions and to ensure that appropriate referrals are made.  To provide a platform for discussion integrating specialist palliative care with respiratory and primary care. | SCDAT: use of the quality improvement theory: Define-measure-analyse-improve-control (DMAIC) methodology.  Tool was adapted from one used with renal dialysis patients and refined by involving members of the ILD, palliative care and psychology teams. Feedback from multidisciplinary discussions was incorporated in tool. | The tool had a positive impact on the assessment of palliative and supportive needs and on referrals for support.  The meeting increases discussions on advanced care planning and on referrals for support |
| NPP (Named patient program)  Chaudhuri 2014  (41) | To manage IPF patients treated with Pirfenidone | NR | The authors highlighted the added value of a specialist nurse in the management of patients. |
| IPF care  Duck 2017  (43) | *“Program was set up to address the unmet need for an initiative that advocates and supports treatment adherence for patients, from initiation of pirfenidone therapy through to longer term exposure.”* | Theoretical basis: NR  Development: collaboration between Intermune and healthcare professionals. | Patients were satisfied with the program and the authors highlighted the opportunity of the program to discuss any issues patients have. |
| Performance improvement study  Fernandez-Perez 2018  (35) | To improve team-based care and health for patients with IPF by improving documentation and processes to better assess and treat patients.   - Identification quality indicators - Improve multidisciplinary team collaboration - Development of sustainable education tools - Improvement patient communication and care - Improvement patient continuity of care | Use of PDSA stepwise approach (cycles)  Involved members: physicians, nurses, medical assistants, physician assistant, professional education team and biostatistics team  Review of current practices and identification of metrics through a blended approach of baseline electronic medical record audits, interviews with team members and peer reviewed literature review. | Changes were implemented based on the needs in the local practice, including improvements in the documentation, adjustments in the medical records, patient education |
| Care coordinator  Hambly 2019  (42) | “*An IPF care coordinator has the potential to improve treatment compliance through early recognition and management of drug related adverse events by providing education, support and empowerment to IPF patients”.* | NR | Benefits were observed (patient satisfaction, costs, physician time) when including a care coordinator in IPF management. |
| Multidisciplinary collaborative care model  Kalluri 2018  Pooler 2018  (38,44) | To meet the care needs of patients and their caregiver regarding palliative care and end-of-life care. | NR | The new approach had a positive impact on the care utilization and on the preferred place of death of the patients.  Bereaved caregivers were satisfied with the approach. |

Legend with abbreviations: **ILD** (interstitial lung diseases), **NR** (not reported), **PRO (**patient-reported outcome), **GORD** (gastro-esophageal reflux disease), **PROM** (patient-reported outcome measure), **FVC** (forced vital capacity), **SCDAT** (supportive care decision aid tool), **DMAIC** (define-measured-analyze-improve-control), **NPP** (named patient program), **AEs** (adverse events), **PDSA** (plan-do-study-act), **HRQoL** (health-related quality of life)
